# Screening for Depressive Mood During Acute Chikungunya Infection in Primary Healthcare Settings

**DOI:** 10.3390/ijerph15112552

**Published:** 2018-11-14

**Authors:** Efrén Murillo-Zamora, Oliver Mendoza-Cano, Benjamín Trujillo-Hernández, Xóchitl Trujillo, Miguel Huerta, José Guzmán-Esquivel, Martha Alicia Higareda-Almaraz, Agustin Lugo-Radillo, Ignacio Moreno-Gutiérrez, Enrique Higareda-Almaraz, Mónica Ríos-Silva

**Affiliations:** 1 Instituto Mexicano del Seguro Social, Departamento de Epidemiología, Unidad de Medicina Familiar No. 19, Av. Javier Mina 301, Col. Centro, CP 28000 Colima, Mexico; efren.murilloza@imss.gob.mx; 2 Programa de Doctorado en Ciencias Médicas, Facultad de Medicina, Universidad de Colima, Av. Universidad 333, Col. Las Víboras, CP 28040 Colima, Mexico; 3 Facultad de Ingeniería Civil, Universidad de Colima, Km. 9 Carretera Colima-Coquimatlán, CP 28400 Coquimatlán, Colima, Mexico; 4 Facultad de Medicina, Universidad de Colima, Av. Universidad 333, Col. Las Víboras, CP 28040 Colima, Mexico; trujillobenjamin@hotmail.com (B.T.-H.); pepeguzman_esquivel@outlook.com (J.G.-E.); 5 Centro Universitario de Investigaciones Biomédicas, Universidad de Colima, Av. 25 de julio 965, Col. Villas San Sebastián, CP 28045 Colima, Mexico; rosio@ucol.mx (X.T.); huertam@ucol.mx (M.H.); 6 Instituto Mexicano del Seguro Social, Unidad de Investigación en Epidemiología Clínica, Av. de los Maestros 148, Col. Centro, CP 28000 Colima, Mexico; 7 Instituto Mexicano del Seguro Social, Jefatura de Servicios de Prestaciones Médicas, Álvaro Obregón 184, Col. Centro, CP 28000 Colima, Mexico; marthahigareda12@hotmail.com (M.A.H.-A.); dr.morenog@hotmail.com (I.M.-G.); 8 CONACYT—Universidad Autónoma Benito Juárez de Oaxaca. Ex Hacienda de Aguilera S/N, Carretera Oaxaca-San Felipe del Agua, CP 68020 Oaxaca, Mexico; alugora@conacyt.mx; 9 Independent Researcher. Manuel Payno 601, Col. Jardines de Vista Hermosa, CP 28017 Colima, Mexico; enriquehigaredaalmaraz@yahoo.com.mx; 10 Universidad de Colima-Cátedras CONACyT, Centro Universitario de Investigaciones Biomédicas, Av. 25 de julio 965, Col. Villas San Sebastián, CP 28045 Colima, Mexico

**Keywords:** depression, primary health care, chikungunya fever, acute disease, patient health

## Abstract

**Background:** We aimed to screen for depressive mood experienced during acute chikungunya (CHIKV) infection, and to evaluate the association of several exposures with the risk of depressive symptoms. **Methods:** A cross-sectional analysis of a multicenter cohort study took place and data from 354 adult individuals with confirmed CHIKV infection were analyzed. Participants were recruited in primary health care settings and the Patient Health Questionnaire-2 (PHQ-2) was used. Prevalence odds ratios (OR) and 95% confidence intervals (CIs) estimated by means of logistic models were used. **Results:** Depressive mood (PHQ-2 score 3 or higher) was reported by 44.1% of individuals. Subjects with articular effusion (OR = 3.37, 95% CI 1.77–8.11), gastrointestinal manifestations (diarrhea, vomiting or abdominal pain, OR = 1.97, 95 CI 1.21–3.19), and higher length of severe arthralgia (reference ≤ 14 days: 15–30 days, OR = 3.38, 95% CI 1.78–6.41; ≥ 30 days, OR = 1.69, 95% CI 0.95–3.01) were more likely to self-report depressive mood. Increasing age (≥ 40 years old, OR = 0.55, 95% CI 0.31–0.95) and rash (OR = 0.54, 95% CI 0.30–0.98) were associated with a decreased risk of depressive mood. **Conclusions:** Depressive mood seemed to be a frequent event among analyzed individuals, and markers associated with its risk were identified.

## 1. Introduction

Chikungunya virus (CHIKV) is an arbovirus transmitted to humans by the bite of infected *Aedes* (*Ae.*) mosquitoes [1]. Massive disease outbreaks have been documented in most tropical and subtropical areas of the world, and primary care plays an important role in the diagnosis, medical management and long-term care of patients with acute CHIKV infection [2,3].

**Figure 1:**
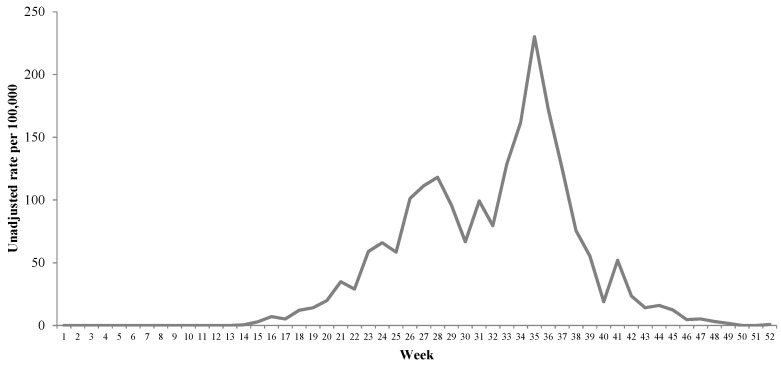
Unadjusted incident rate (per 100,000) of chikungunya virus infection among affiliates to the Mexican Institute of Social Security (*IMSS* from its Spanish acronym) in the state of Colima, Mexico, 2015.

The state of Colima, located in the western region from Mexico, is a subtropical area where the permanent presence of *Ae. aegypti* has been documented. The first outbreak of CHIKV infection took place in Colima during 2015 and, as observed in Figure 1, high incidence rates were observed. The attack rate is unknown due to the lack of seroprevalence surveys but the attack rates among other populations range from 37% to 75% [4,5,6,7,8].

Acute illness is characterized by profound articular pain and most of cases are self-limited and disappear within 7–10 days [9]. However, numerous acute complications have been described and include an increased risk of psychiatric disorders, namely depressive mood [10,11]. Scientific knowledge regarding psychiatric CHIKV-associated comorbidity is limited; it may potentially worsen the course of acute disease by affecting the social functionality of CHIKV patients [12]. The evaluation of markers associated with the risk of depressive symptoms during acute disease may be useful, in primary care settings, to identify patients with an increased risk of psychiatric disorders and to improve their medical prognosis.

We aimed to assess the prevalence of depressive mood experienced during acute CHIKV infection among adult individuals. In addition, the association of several markers with the risk of depressive symptoms was evaluated.

## 2. Materials and Methods

### 2.1. Study Design

A cross-sectional analysis from a multicenter cohort study took place in the state of Colima, Mexico, from October 2017 to February 2018. The medical units (*n* = 3) where the individuals were recruited are urban primary healthcare facilities and belong to the Mexican Institute of Social Security (*IMSS* from its Spanish acronym).

### 2.2. Participants

Individuals aged 15 years and older with confirmed cases of CHIKV infection and acute disease onset from June to December 2015 were enrolled. Diagnostic criteria for CHIKV involved (1) the abrupt onset of severe (incapacitating) arthralgia or arthritis after autochthonous transmission was documented, or (2) laboratory evidence (reverse transcription quantitative-polymerase chain reaction, RT-qPCR) of infection among symptomatic cases. Subjects with personal history of systemic rheumatic diseases (multiple sclerosis, rheumatoid arthritis, or systemic lupus erythematosus) were excluded. A complete description of the selection criteria was previously published [13] and 354 subjects were enrolled. CHIKV infection was laboratory-confirmed in 61.3% (*n* = 217) of participants.

### 2.3. Data Collection

Subjects were interviewed at six months from the acute disease onset by standardized health professionals (practicing family physicians). A structured questionnaire was employed to collect the data of interest and included demographic variables, personal history of chronic non-communicable diseases, and the clinical characteristics of acute illness. The questionnaire was designed for the purposes of the cohort study [13]. The Patient Health Questionnaire-2 (PHQ-2) tool was used to screen for depressive symptoms experienced during the acute disease and a score of 3 or higher was considered as positive for depressive mood [14].

### 2.4. Laboratory Methods

The venous blood samples from the study subjects were obtained within 5 days of illness onset and RT-qPCR analysis (QuantiTect^®^, QIAGEN, Velo, The Netherlands) was performed. The analytical procedure took place in the Central Laboratory of Epidemiology (*IMSS*, Mexico City, Mexico).

### 2.5. Statistical Analysis

Summary statistics were estimated. Prevalence odds ratios (OR) and 95% confidence intervals (CI), estimated by means of unconditional logistic regression models, were used to evaluate the association of the measured exposures with the presence of depressive symptoms on acute illness.

**Ethical considerations:** This study was approved by the National Commission of Clinical Research from the IMSS (approval R-2016-785-004). All participants signed a written informed consent prior to being interviewed and the practicing family physicians that performed data collection signed a confidentiality agreement. To guarantee the anonymity of the research participants, all the identification variables were omitted from the database and in their place an alphanumeric code was assigned to each individual.

## 3. Results

The mean interval between disease onset and the date of the structured interview was 182.1 ± 4.6 days and the mean age at diagnosis of acute infection was 41.2 ± 15.2 years old. The characteristics of study sample for selected variables are shown in Table 1. Most of the participants were female (67.8%).

The prevalence of depressive mood was 44.1%. During the acute symptomatic infection, statically significant differences were observed among groups (PHQ-2 score <3 vs. ≥3) in terms of self-reported articular effusion at any site (73.4% vs. 93.6), fatigue (92.3% vs. 98.7%), gastrointestinal manifestations (diarrhea, vomiting or abdominal pain; 32.3% vs. 48.7%), number of arthralgia sites (≥8 affected joints; 42.4% vs. 56.4%), and length (days) of severe articular pain at acute disease. No significant differences were documented in terms of sex, age, diagnostic criteria of infection or self-reported history of chronic non-communicable diseases (Table 1).

The associations of the evaluated exposures with the risk of depressive symptoms during acute cute illness are shown on Table 2. In multiple analysis, an increased risk was observed among individuals with self-reported articular effusion at any site (OR = 3.37, 95% CI 1.77–8.11), gastrointestinal clinical manifestations (OR = 1.97, 95% CI 1.21–3.19), and length of severe (incapacitating) arthralgia (≤14 days as compared to 15–30 days, OR = 3.38, 95% CI 1.78–6.41; ≥30 days, OR = 1.69, 95% CI 0.95–3.01). Two exposures showed a significant decreased frequency of depressive symptoms: age ≥ 40 years old (when compared with individuals younger than 40 years old: OR = 0.55, 95% CI 0.31–0.95) and the presence of rash (OR = 0.54, 95% CI 0.30–0.98).

## 4. Discussion

Our findings suggest that acute CHIKV infection impacts negatively on non-biological dimensions of health and depressive mood (score of 3 or higher on PHQ-2) was observed in more than two fifths of analyzed individuals. Several factors were associated with the risk of depressive symptoms; these results may be used in primary healthcare settings to identifying patients in whom a multidimensional impact of the infection is plausible.

The PHQ-2 does not provide a psychiatric diagnosis but is a brief and effective tool but provides exploratory data regarding the impact of a highly incapacitating disease on mental health. Published studies evaluating the frequency of CHIKV-associated depressive symptoms during acute infection are scarce. In a study that took place in India and where data from 20 subjects were analyzed, the prevalence of depressive disorders at acute illness was 15% [5]. Our study, in our best knowledge, is the first screening for depressive mood in a large study sample. Among patients with rheumatic illness, a high incidence of mood disorders has been observed [15].

Depressive disorders in acute care are complex and multifactorial events [16,17]. In addition, the perception of pain may be exacerbated by depression and other mood disorders [18]. In our study, self-reported articular effusion (at any site) during acute illness (OR = 3.37, 95% CI 1.77–8.11) and gastrointestinal manifestations (diarrhea, vomiting or abdominal pain; OR = 1.97, 95% CI 1.21–3.19) were associated with and increased risk of depressive mood. We hypothesize that joint edema may increase the perceived disability and therefore have a negative impact on mental health; no significant differences were documented in the risk of depressive mood in terms of self-reported pain severity (severe pain, numeric rating scale (NRS) ≥7; OR = 1.08, 95% CI 0.67–1.72).

The presence of gastrointestinal illness, given that acute CHIKV infection is characterized by profound arthralgia and impaired ambulation, may also worsen the perceived impact of this arboviral disease.

Increasing age in our study seems to be inversely associated with the risk of depressive mood (age 40 years or older, OR = 0.55, 95% CI 0.31–0.95), even after the adjustment by personal history of chronic non-communicable diseases. This finding may be secondary to a higher impact of severe pain and impaired ambulation in younger patients. During the 2015 disease outbreak in the state of Colima, Mexico, most of the registered cases (nearly 75%) occurred among individuals aged 20–59 years, who are more likely to be employed. Interestingly, the presence of rash seemed to be a protective factor for the presence of depressive mood and, if this observation is later reproduced among other populations, the pathogenic mechanism should be elucidated.

Self-reported fatigue was associated with a significant increased risk of depressive in univariate analysis (OR = 5.86, 95% CI 1.31–26.18) but not in multiple analysis (OR = 4.39, 95% CI 0.90–21.44), even when its prevalence was high (95.5%). Fatigue is a common symptom of depressive mood [19] and the association of CHIKV illness with the risk of chronic fatigue was recently documented [20].

The current scientific knowledge supports the benefits of screening for mood disorders in adult individuals in primary care [21]. An accurate diagnosis, effective medical management, and appropriate follow-up must be ensured [22]. However, the viability and effectiveness of depression screening in primary care settings has been questioned [23].

The limitations of a cross-sectional study must be considered in the interpretation of our results (i.e., temporal ambiguity and recall bias). Other limitations should be considered. First, laboratory testing was not available for 38.7% of analyzed individuals and they were enrolled according to the fulfillment of clinical and epidemiological data. The state of Colima, Mexico, is a subtropical area with the permanent presence of *Ae. aegypti* [24,25]. According to normative standards, molecular assays are performed on 5% of randomly-selected suspected cases of acute CHIKV infection. However, in our best knowledge, CHIKV illness is the only viral infection characterized by profound articular pain [26]. During a similar outbreak in the state of Chiapas, Mexico, CHIKV was isolated in nearly 80% of blood samples from febrile patients [27]. When study subjects with laboratory-confirmed infection were compared with those who lacked of laboratory evidence of the pathogen, no significant differences were observed in terms of PHQ-2 score (score ≥3, 44.7% vs. 43.1%, *p* = 0.763). Differences in terms of gender, age, and prevalence of chronic non-communicable diseases were not significant (data not presented).

Second, only one screening tool (PHQ-2) was employed to screen for mood disorders. The cited instrument was chosen given its simplicity and good sensitivity and specificity in detecting individuals with a higher risk for major depression [14], also in family medicine practice [28]. Third, an unknown proportion of adults classified as positive for depressive mood during acute infection may have had this mood disorder prior to the arboviral infection. However, the overall lifetime prevalence of depressive mood among Mexican individuals is considerable lower than in our estimate (9.2% vs. 44.1%) [29].

Fourth, since a high prevalence (>10%) of the binary outcome was documented, the computed OR may be overestimated [30,31]. Fifth, a limited number of exposures were evaluated given the particular design of the study, and other contextual variables were not measured. The current situation of Mexico is complex and, resulting from the operation of drug trafficking groups and other related problems, an increase in violent events has been observed and they may impact on mental health in the population [32].

Sixth, study sample was integrated by patients of only one healthcare institution and may not be fully representative of the source population. The *IMSS* has 11 primary healthcare facilities strategically located along the state; 45% from the total population of the state is affiliated to its medical services [33] and the sociodemographic profile of insured individuals is heterogeneous.

## 5. Conclusions

The depressive mood seemed to be a frequent event among analyzed individuals and markers associated with its risk were identified. Primary care plays a fundamental role in the screening of mood disorders and in the treatment and management of CHIKV patients and, if later replicated, our findings may be useful to identify patients with an increased risk of multidimensional impacts of the acute disease.

## Figures and Tables

**Table 1:** Baseline characteristics of study sample and Patient Health Questionnaire-2 (PHQ-2) score during acute chikungunya virus infection, Mexico 2015.

	Overall		PHQ-2 score				*p*
	*n* (%)		<3, *n* (%)		≥3, *n* (%)		
Total	354		198		156		
Sex							
Male	114	(32.2)	70	(35.3)	44	(28.2)	0.153
Female	240	(67.8)	128	(64.7)	112	(71.8)	
Age (years)							
<40	174	(49.2)	90	(45.5)	84	(53.8)	0.117
≥40	180	(50.8)	108	(54.5)	72	(46.2)	
Diagnostic criteria ^a^							
Clinical and epidemiological data	137	(38.7)	78	(39.4)	59	(37.8)	0.763
Laboratory-positive	217	(61.3)	120	(60.6)	97	(62.2)	
*Self-reported history of:*							
Type 2 diabetes mellitus (yes)	46	(13.0)	24	(12.1)	22	(14.1)	0.582
Arterial hypertension (yes)	74	(20.9)	46	(23.3)	28	(18.0)	0.225
Osteoarthritis (any site, yes)	38	(10.7)	22	(11.1)	16	(10.3)	0.796
*At acute disease:*							
Fever (yes)	322	(91.0)	178	(89.9)	144	(92.3)	0.433
Articular effusion (any site, yes)	292	(82.5)	146	(73.4)	146	(93.6)	<0.001
Rash (yes)	282	(79.7)	164	(82.8)	118	(75.6)	0.095
Headache (yes)	316	(89.3)	176	(88.9)	140	(89.7)	0.796
Fatigue (yes)	338	(95.5)	184	(92.3)	154	(98.7)	0.009
Gastrointestinal manifestations (yes) ^b^	140	(39.5)	64	(32.3)	76	(48.7)	0.002
Arthralgia sites (*n*)							
<8	182	(51.4)	114	(57.6)	68	(43.6)	0.009
≥8	172	(48.6)	84	(42.4)	88	(56.4)	
Severity of articular pain ^c^							
Mild-moderate	28	(7.9)	20	(10.1)	8	(5.1)	0.085
Severe	326	(92.1)	178	(89.9)	148	(94.9)	
Length of severe arthralgia (days)							
1–14	158	(44.6)	106	(53.5)	52	(33.3)	<0.001
15–30	82	(23.2)	32	(16.2)	50	(32.1)	
30 or more	114	(32.2)	60	(30.3)	54	(34.6)	

The *p*-value from chi-squared tests is presented. ^a^ The diagnostic criteria of acute chikungunya infection included the abrupt onset of severe (incapacitating) polyarthralgia after the autochthonous transmission of the virus was documented or laboratory evidence (real-time reverse transcription polymerase chain reaction, RT-qPCR) in venous blood samples from symptomatic individuals. ^b^ The gastrointestinal manifestations included the presence of diarrhea, vomiting, or abdominal pain. ^c^ An auto-declarative numeric rating scale (NRS) ranging from 0 to 10 was used (mild-moderate pain, < 7 points; severe pain, ≥ 7 points).

**Table 2:** Markers associated with the risk of depressive symptoms (Patient Health Questionnaire-2 score ≥3) during the acute chikungunya virus infection, Mexico 2015.

	Univariate Analysis			Multiple Analysis		
	OR (95% CI)		*p*	OR (95% CI)		*p*
Sex						
Male	1.00			1.00		
Female	1.39	(0.88–2.19)	0.154	1.02	(0.61–1.70)	0.940
Age (years)						
<40	1.00			1.00		
≥40	0.71	(0.47–1.09)	0.117	0.55	(0.31–0.95)	0.031
Self-reported history of any chronic non-communicable disease ^a^						
No	1.00			1.00		
Yes	0.76	(0.49–1.20)	0.244	1.11	(0.62–1.97)	0.732
*At acute disease:*						
Articular effusion (any site)						
No	1.00			1.00		
Yes	5.20	(2.54–10.62)	<0.001	3.37	(1.77–8.11)	0.001
Rash						
No	1.00			1.00		
Yes	0.64	(0.38–1.08)	0.097	0.54	(0.30–0.98)	0.042
Fatigue						
No	1.00			1.00		
Yes	5.86	(1.31–26.18)	0.021	4.39	(0.90–21.44)	0.067
Gastrointestinal manifestations ^b^						
No	1.00			1.00		
Yes	1.99	(1.29–3.07)	0.002	1.97	(1.21–3.19)	0.006
Arthralgia sites (n)						
<8	1.00			1.00		
≥8	1.76	(1.15–2.68)	0.009	1.33	(0.83–2.11)	0.235
Severity of articular pain ^c^						
Mild–moderate	1.00			1.00		
Severe	1.44	(0.94–2.20)	0.091	1.08	(0.67–1.72)	0.759
Length of severe arthralgia (days)						
1–14	1.00			1.00		
15–30	3.19	(1.83–5.54)	<0.001	3.38	(1.78–6.41)	<0.001
30 or more	1.83	(1.12–3.01)	0.016	1.69	(0.95–3.01)	0.071

The prevalence Odds ratios (OR) and 95% confidence intervals (CIs) estimated by means of unconditional logistic regression models are presented. ^a^ Type 2 diabetes mellitus, arterial hypertension and osteoarthritis are included. ^b^ The gastrointestinal manifestations included the presence of diarrhea, vomiting, or abdominal pain. ^c^ An auto-declarative numeric rating scale (NRS) ranging from 0 to 10 was used (mild–moderate pain, < 7 points; severe pain, ≥ 7 points).
